# Systems for Targeted Silencing of Gene Expression and Their Application in Plants and Animals

**DOI:** 10.3390/ijms25105231

**Published:** 2024-05-11

**Authors:** Daria M. Motorina, Yuliya A. Galimova, Nadezhda V. Battulina, Evgeniya S. Omelina

**Affiliations:** Institute of Molecular and Cellular Biology, Siberian Branch of the Russian Academy of Sciences, 630090 Novosibirsk, Russia

**Keywords:** gene silencing, optogenetics, CRISPR/Cas, RNAi, TALE, zinc finger protein

## Abstract

At present, there are a variety of different approaches to the targeted regulation of gene expression. However, most approaches are devoted to the activation of gene transcription, and the methods for gene silencing are much fewer in number. In this review, we describe the main systems used for the targeted suppression of gene expression (including RNA interference (RNAi), chimeric transcription factors, chimeric zinc finger proteins, transcription activator-like effectors (TALEs)-based repressors, optogenetic tools, and CRISPR/Cas-based repressors) and their application in eukaryotes—plants and animals. We consider the advantages and disadvantages of each approach, compare their effectiveness, and discuss the peculiarities of their usage in plant and animal organisms. This review will be useful for researchers in the field of gene transcription suppression and will allow them to choose the optimal method for suppressing the expression of the gene of interest depending on the research object.

## 1. Introduction

Gene expression is an essential step in the implementation of genetic information. The temporal and spatial regulation of the gene expression process is known to be responsible for the fate and function of different cells and tissues, homeostasis, development, environmental adaptation, and the behaviour of complex multicellular organisms. Disruptions in gene expression can result in various pathologies, including congenital anomalies, developmental defects, and oncological diseases [1,2]. 

**Figure 1:**
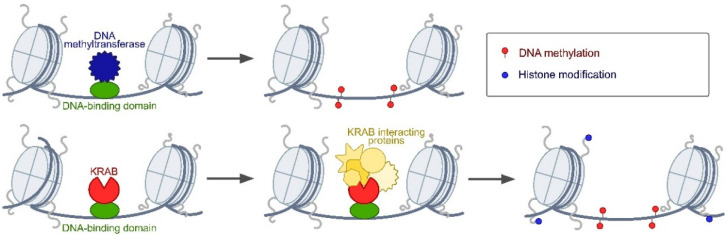
Scheme of gene silencing mediated by chimeric repressor proteins. These proteins consist of a DNA-binding domain and a repressor domain, e.g., DNA methyltransferase (top panel) and Krüppel-associated box (KRAB) domain (bottom panel).

Currently, there are many different approaches to the regulation of gene expression [3,4]. However, most approaches are devoted to the activation of transcription, and the methods of targeted gene silencing are much fewer in number. At the beginning of the century, RNA interference (RNAi) was the most popular method for disrupting gene expression in different organisms, including plants and animals. Later, other approaches, including zinc finger proteins (ZFPs) and transcription activator-like effectors (TALEs), became available. These approaches utilize artificial DNA-binding domains that recognise specific DNA sequences. Other systems for targeted gene silencing, including chimeric transcription factors and the more recent CRISPR interference (CRISPRi) and optogenetic tools, are based on the fusion of the studied protein with different repressor domains. In animals, the most often used repressor domains include the Krüppel-associated box (KRAB), which is a 75 amino acid transcriptional repressor domain commonly found in eukaryotic ZFPs, and the catalytic domains of the DNA methyltransferases (e.g., DNMT3a, DNMT3b) [5,6,7,8,9] (Figure 1). In addition to these domains, 195 repressor domains were recently identified in known repressors and in proteins not previously associated with gene silencing in *Drosophila melanogaster* [10]. In plants, the EAR repression domain (SRDX), consisting of a 12 aa sequence LDLDLELRLGFA, is most often used for gene silencing [11]. Controlled gene silencing approaches are most often used for defining a gene’s function. However, they also can be used in the medical field to treat various genetic disorders. In this paper, we compare various systems for targeted gene silencing in plants and animals, such as RNAi, chimeric transcription factors, chimeric ZFPs, TALE-based repressors, optogenetic tools, and CRISPR/Cas-based repressors. 

## 2. Systems for Targeted Gene Silencing

### 2.1. RNA Interference

**Figure 2:**
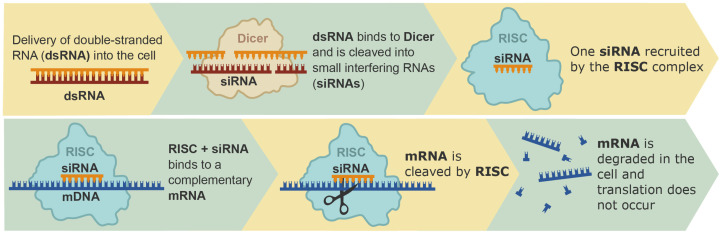
Scheme of the RNA interference (RNAi) process. After the delivery of double-stranded RNA (dsRNA) into the cell, the dsRNA is further cleaved into small interfering RNAs (siRNAs) by the ribonuclease Dicer. Then, siRNA binds with the RNA-induced silencing complex (RISC). RISC uses this siRNA to establish homologous RNAs in the cells, which triggers the endo-nucleolytic cleavage and translational inhibition of the cognate target mRNA, thereby leading to gene silencing.

RNA interference (RNAi) is a mechanism of gene regulation involving double-stranded RNA (dsRNA) molecules that are complementary to the gene sequence. Repression is achieved by reducing the number of transcripts through RNA degradation processes. The RNAi process occurs in several stages: initially, dsRNA that is complementary to the target gene’s transcript sequence is processed by an evolutionary-conserved type III ribonuclease called Dicer, which converts dsRNA into small interfering RNAs (siRNAs) of 21–25 bp length [12,13] (Figure 2). These siRNAs are then incorporated into the multiprotein RNA-induced silencing complex (RISC), which contains a member of the Argonaute protein family, providing binding with the siRNA and positioning it in a conformation that facilitates target recognition [14,15]. RISC unwinds the siRNA and this complex associated with the antisense siRNA strand is directed to the homologous gene transcript. RISC subsequently cleaves and degrades the RNA target, thereby leading to gene silencing [16]. Then, the RISC-siRNA complex targets another mRNA transcript and the process is repeated.

#### 2.1.1. Application of RNAi in Plants

The phenomenon of RNAi was first observed as early as 1990 by the researchers Napoli and Jørgensen [17]. These scientists attempted to overexpress the *chalcone synthase* (*CHS*) gene in pigmented petunia petals by introducing a chimeric gene of petunia, *CHS*. The *CHS* gene encodes an enzyme involved in flavonoid biosynthesis, including the production of the anthocyanin pigments in petunia petals. Unexpectedly, the introduced gene reduced anthocyanin biosynthesis, resulting in the formation of totally white and/or patchy flowers with white areas on the pigmented petals [17]. This phenomenon was not well understood, and the authors of this study named it co-suppression [18] and linked it with the presence of sense or antisense RNA, rather than dsRNA. 

Currently, RNAi is actively used in plants. The main approaches to RNAi in plants are Virus-Induced Gene Silencing (VIGS), Host-Induced Gene Silencing (HIGS), and Spray-Induced Gene Silencing (SIGS).

#### Virus-Induced Gene Silencing (VIGS)

RNAi in plants is often carried out using VIGS [19]. VIGS is a highly effective method that uses an RNA-mediated mechanism of antiviral defence. Van Kammen first introduced the term VIGS to characterise the phenomenon of “recovery from viral infection” [20]. In plants infected with unmodified viruses, VIGS targets the viral genome [21]. However, when using viral vectors carrying host gene inserts, the process can be directed against the corresponding mRNA. Most often, VIGS vectors are standard binary Ti plasmid-derived vectors used for *Agrobacterium tumefaciens*-mediated plant transformation [22]. A multiple cloning site is added within the virus genome to insert a fragment of the endogenous host plant target gene. Typically, this fragment is a sequence of the middle part of the mRNA, with a length of 200 to 1300 bp [23]. T-DNA containing the viral genome is then integrated into the host genome through the process of agroinfiltration. Viral RNAs can form dsRNAs. To increase dsRNA formation, direct inverted repeats have been added to some vector systems [19]. Furthermore, VIGS is almost identical to classical RNAi. dsRNAs are recognised by Dicer-like proteins [19]. These proteins convert dsRNA into siRNAs, which form complexes with the Argonaute proteins and target their corresponding RNA, resulting in gene silencing by the endonucleolytic slicing of transcripts [24]. In addition to agroinfiltration, other methods of delivering the viruses are used in VIGS experiments: the infection of plants with in vitro transcribed viral RNA and the infection of plants with viral particles from infected donor plants [19]. The first VIGS vector was constructed using Tobacco mosaic virus to inhibit the synthesis of carotenoids in *Nicotiana benthamiana* leaves by the suppression of the *phytoene desaturase* (*PDS*) gene’s expression [25].

VIGS is widely used to study plant gene functions under abiotic stress (such as drought [26], salt stress [27], chilling stress [28]) and during development [29]. In addition, RNAi has become an effective tool for studying the function of plant genes under the biotic stresses caused by various pests, e.g., whiteflies [30], and for crop protection from pathogens, such as insects, viruses, and fungi [31,32,33,34,35]. RNAi, in addition to model plants, is also used in fruit trees [36,37]. 

VIGS is a promising and efficient method for studying plant gene function that differs from other tools due to its ability to rapidly generate phenotypes without the need for stable transformation. This method is more affordable in terms of cost than other methods such as transposon insertion and genome editing. However, the VIGS method has some limitations: first, off-target silencing [38]. Second, the effects of the reduced gene expression caused by VIGS may be different in various parts of the plant [39]. Therefore, to accurately assess the effects of a gene subjected to VIGS, it is necessary to screen the phenotypes of a large number of plants to ensure that the results from a few plants are representative of the entire organism. In addition, changes in environmental conditions, including light intensity, temperature, and humidity, can affect the effectiveness of this silencing [40,41,42].

#### Host-Induced Gene Silencing (HIGS)

HIGS is recognised as a potent pesticide-free alternative to chemical treatments for the protection of plants from pests [43]. It involves employing homologous dsRNAs or artificial microRNAs (amiRNAs) in host plants to silence genes in pathogens and pests [44]. The first induction of RNAi using RNAs produced in plants was observed in the free-living nematode *Caenorhabditis elegans* [45]. To achieve this, *N. benthamiana* transgenic plants expressing GFP were generated. RNA extracts from plants, in which GFP expression had been spontaneously silenced, were injected into a GFP-expressing strain of *C. elegans*, resulting in the RNAi of the *GFP* transgene. RNA extracts from non-silenced plants did not influence the GFP expression in the worms.

In 2006, Huang et al. demonstrated the use of HIGS to suppress, in root-knot nematodes (RKNs), the parasitism gene *16D10,* which mediates infection and parasitism [46]. The utilisation of in vitro and in vivo *16D10* dsRNA resulted in the silencing of the parasitism gene *16D10* in RKNs. In vivo experiments in *Arabidopsis* plants enabled their effective resistance against the four major RKN species. Currently, numerous studies are devoted to the application of HIGS in agricultural plants for their protection from various pathogens and pests [47,48,49,50].

#### Exogenous RNAi and Spray-Induced Gene Silencing (SIGS)

In plant biotechnology, the exogenous use of dsRNA for RNAi-mediated silencing, the so-called exogenous RNAi (exo-RNAi), is possible. There are a variety of application methods for the delivery of exogenous RNAs (dsRNAs/siRNAs/short hairpin RNAs (shRNAs)) into plants, such as infiltration, root/seed soaking, and mechanical inoculation [51,52]. One of the most promising methods for its large-scale use in agricultural crops is spraying [53]. The potential for the external application of dsRNA, by spraying on plant leaves, to induce the RNAi-dependent inhibition of pathogenic fungi’s growth was demonstrated by Koch et al. in 2016 and named SIGS [54]. Around the same time, Wang et al. showed that the treatment of surfaces of fruits, vegetables, and flowers with external dsRNAs targeting the *Dicer*-like genes of *Botrytis* *cinerea* fungi inhibits grey mould disease [55]. The discovery that sprayed dsRNA penetrates plant cells [54] made it possible to use SIGS not only against fungi genes, but also against viruses and plant genes. At present, the effectiveness of some plants’ protection spraying them with dsRNAs and siRNAs targeting the key genes of pathogenic fungi and viruses is well established [54,56,57,58,59,60]. Exogenously induced silencing has also been successfully used for plants’ gene suppression to improve their drought resilience [61] and affect their anthocyanin accumulation [62,63]. The mechanisms of the systemic transport of spray-applied dsRNA in plants are still largely not understood (some details are reviewed in [53]). To improve its practical application and increase dsRNA survivability and uptake, researchers use different RNA carriers, such as polymeric, lipid-based, and inorganic nanoparticles [60,64,65,66].

It should be noted that while the exogenic application of dsRNA counts as alternative to genome-modified methods, the usage of exo-RNAi against plant genes in addition to mRNA degradation may also trigger the RNA-directed DNA methylation (RdDM) of cognate DNA sequences and affect the epigenome [67,68,69].

#### 2.1.2. Application of RNAi in Animals

Initially, the use of the RNAi method was limited to flies, worms, and plants, with powerful and specific repression not achievable in mammals [70,71]. This was explained by the fact that introducing long dsRNAs into mammalian cells induces an interferon response, which triggers a general translation inhibition, nullifying the RNA sequence’s specificity [72]. In 2001, RNAi was successfully carried out in different mammalian cell lines, including human HEK293 and HeLa cells, for the first time [73]. By 2003, this method was already being applied to various cell lines and even whole organisms [74,75,76,77,78,79].

**Figure 3:**
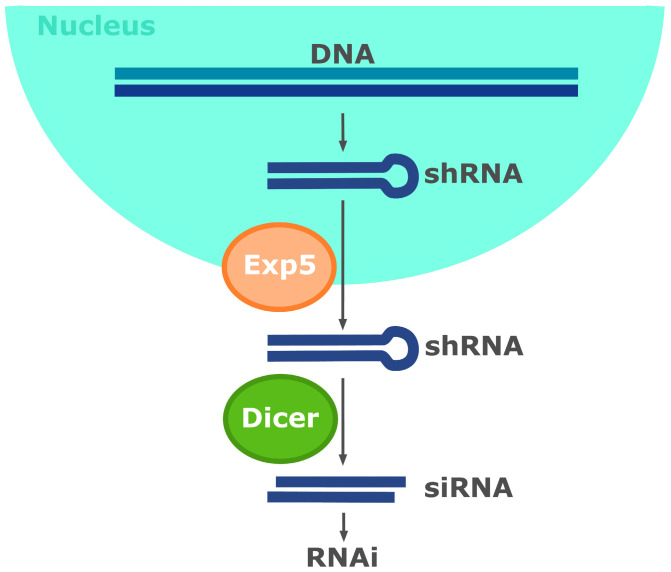
Scheme of the cellular processing of short hairpin RNAs (shRNAs). shRNAs are expressed within the nucleus, where they form hairpins. shRNAs are transported from the nucleus to the cytoplasm by Exportin 5 (Exp5), and they are then cleaved by Dicer into siRNA and join the RNAi pathway.

The widespread adoption of the method was facilitated by the fact that siRNAs are relatively easily and quickly chemically synthesised. Commercial manufacturers have begun offering the synthesis of siRNA oligonucleotides, making the RNAi method more attractive. Additionally, siRNAs can be endogenously expressed in the form of short RNAs with a stem–loop structure (short hairpin RNA, shRNA), which can be used to decrease the expression of the target gene [80]. Usually, shRNA is delivered into cells using plasmids or viral/bacterial vectors [80,81]. In contrast to siRNA-mediated RNAi, which typically lasts 3–5 days in cell cultures, the stable integration of shRNA expression vectors can provide enduring protein repression [82]. The transcription of shRNA can be regulated using inducible promoters [83,84,85]. Moreover, constructing a vector with shRNAs may be less costly than the chemical synthesis of siRNAs, and the targeting of specific tissue types can be achieved through the use of cell- or tissue-specific promoter sequences [86,87,88]. The cellular processing of shRNA occurs as follows: shRNAs are expressed within the nucleus, where they spontaneously form hairpins. Next, shRNAs are transported from the nucleus to the cytoplasm via Exportin 5 (Exp5) [89]. Subsequently, shRNAs are cleaved by Dicer into active siRNAs, which join the RNAi pathway (Figure 3).

RNAi has been the primary tool for targeted gene repression for many years [90,91]. RNAi-mediated gene silencing has become a promising antiviral approach to protecting humans from infections and developing RNAi drugs [92,93]. However, it is necessary to note that the RNAi method is often not efficient enough and only leads to a slight decrease in the quantity of the transcripts of the gene of interest, ultimately reducing the amount of protein by only 1.5–2 times and having no significant phenotypic effect. Additionally, the RNAi method has drawbacks that are associated with its off-target effects, which can lead to a misinterpretation of the results, especially in the context of large-scale screenings [94,95,96].

An alternative strategy for targeted gene silencing is to use various DNA-binding proteins to recruit repressive factors to the gene of interest. DNA-binding proteins found in nature and artificially obtained (such as galactose-responsive transcription factor Gal4, the tetracycline repressor (TetR), different transcription factors, and proteins containing zinc finger domains) have been fused with transcription repressors and employed for targeted gene repression [97,98,99,100]. In this review, we will consider some of these systems.

### 2.2. The Chimeric Transcription Factors

#### 2.2.1. The Gal4/UAS System

The Gal4/UAS system is a method for studying gene expression and functionality based on the discovery that the binding of the yeast transcription factor Gal4 to the upstream activation sequence (UAS) activates gene expression [101,102,103]. Currently, the Gal4/UAS system is a powerful tool for studying gene expression [104,105,106,107,108].

**Figure 4:**
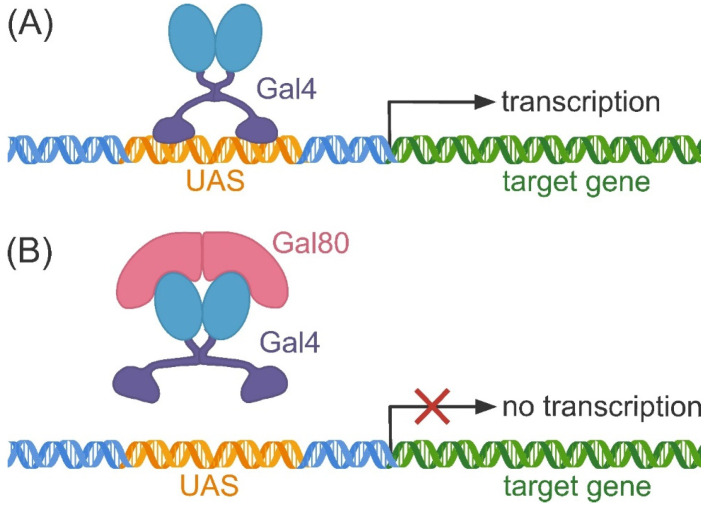
Scheme of the Gal4/UAS system. (**A**) Binding of the Gal4 protein to UAS induces the transcription of the Gal4 reporter gene. (**B**) The Gal80 protein acts as an inhibitor of Gal4 by binding to it and preventing interactions between Gal4 and UAS.

The primary strategy for repressing target genes using the Gal4/UAS system involves the use of the transcriptional repressor Gal80 [109,110,111,112]. Gal80 counteracts Gal4 by directly binding with the amino acids of the Gal4 C-terminal activation domain, preventing an interaction between Gal4 and the transcription machinery [113,114,115]. This prevents the expression of the transgene under the control of UAS sites (Figure 4). For temporal gene expression control, there are two main types of induced Gal80 constructs: a temperature-sensitive variant of Gal80 (Gal80^ts^) and tetracycline-regulated Gal80 (Tet-off Gal80). The first variant expresses Gal80 under the control of the ubiquitous promoter of the *tubulin 1a* gene in *Drosophila*. At 19 °C, Gal80^ts^ is active and capable of suppressing Gal4-mediated expression; at 30 °C, this repression is reduced [111]. The Tet-off Gal80 system contains two promoters, with Gal80 controlled by the TetO promoter and a ubiquitous promoter for regulating tetracycline transactivator molecule (tTA) expression [116]. In the absence of tetracycline, tTA binds to TetO and activates Gal80 expression; in the presence of tetracycline, tTA is inactive, resulting in no Gal80 expression. Therefore, the gene expression mediated by Gal4 can be temporarily controlled by adding tetracycline to the diet. In a recent study using two Gal4 drivers, DJ694-Gal4 (oenocytes) and Mef2-Gal4 (muscle), a comparative analysis of the repression ability of the Gal80^ts^ and Tet-off Gal80 transgenes at different *Drosophila* ages was performed [117]. It was demonstrated that their repressive ability was more dependent on the choice of the Gal4 driver than on the level of system expression and that neither transgene completely blocked gene expression at all ages. However, of both drivers, Gal80^ts^ was more effective, resulting in lower residual expression than Tet-off Gal80, which may be due to the better stability of Gal80^ts^ at low temperatures [117].

One more strategy for Gal4-mediated gene repression is the fusion of the Gal4 DNA-binding domain with different repressor domains, resulting in the generation of chimeric transcription factors.

#### Application of the Gal4/UAS System in Plants

Using a transient expression assay in co-bombarded leaves of *Arabidopsis thaliana*, it was shown that the Gal4 DNA-binding domain, fused with a plant-specific B3 transcription factor At2g36080, reduced the expression of the reporter *Luciferase* (*Luc*) gene [118]. A deletion analysis of the At2g36080 protein sequence has indicated that its repressive activity was due to a short 15 aa B3 repression domain (BRD). The fusion of the transcription factors Shoot meristemless (STM), Agamous (AG), and Cup-shaped cotyledon 2 (Cuc2) with BRD converts them into chimeric dominant repressors in transgenic *Arabidopsis* plants. An analysis of other transcription factors similar to At2g36080 has shown BRD-like repression sequences, which acted as repression domains when fused with the Gal4 DNA-binding domain [118].

The repression of the *β-glucuronidase* (*GUS*) reporter gene in *A. thaliana* was achieved by the fusion of the histone deacetylases HD2A, HD2B, and HD2C with the Gal4 DNA-binding domain. All these chimeric repressors strongly suppressed *GUS* activity compared to controls in transient expression assays of tobacco *N. benthamiana* leaves and in the F1 *Arabidopsis* hybrid plants generated by crossing the *Arabidopsis GUS* reporter line with the Gal4-HD2A effector line [119,120].

#### Application of the Gal4/UAS System in Animals

In *Drosophila*, the Gal4/UAS system has been widely used due to the availability of a large number of Gal4 driver lines covering a broad array of cell- and tissue-specific expression patterns (https://bdsc.indiana.edu/stocks/gal4/index.html, accessed on 6 May 2024) and UAS transgene reporter lines (https://bdsc.indiana.edu/stocks/uas/index.html, accessed on 6 May 2024). To enable the use of the currently available Gal4 driver and UAS reporter lines, a temperature-sensitive Gal80 (Gal80^ts^) protein has been developed in the temporal and regional gene expression targeting (TARGET) system [111,121]. The transcription activation of UAS transgenes by Gal4 was inhibited by Gal80^ts^ at 18 °C, and it was relieved by raising the temperature to 29 °C. However, this system is not perfect, since temperature is known to have a direct impact on the physiology of living systems, including their behaviour, neuropathy, and ageing [122].

To overcome the limitations of the previously described temperature-sensitive system, chemically induced Gal80 systems were developed [116]. Additionally, a drug-stabilizable Gal80 variant has recently been engineered by fusing Gal80 to Destabilizing Domains (DDs) [123]. This system controls the post-translational level of Gal80-DD protein abundance in combination with the specific stabilizing drug Trimethoprim (TMP). In the absence of this drug, the DD unfolds, resulting in the degradation of the Gal80-DD protein and activation of Gal4/UAS transgene expression. In the presence of TMP, the DD properly folds, leading to the inhibition of Gal4 activity by Gal80 and the turning off of the reporter’s expression. This system demonstrated an effective TMP-induced repression of the Gal4/UAS reporter genes in a *Drosophila* cell culture S2R+ and in transgenic flies. Furthermore, this system was combined with CRISPR/Cas9 for the TMP-mediated control of the endogenous *even-skipped* (*eve*) gene.

Gal4/UAS was also successfully combined with the auxin-degron system in *Drosophila* [124]. The auxin-degron system provides the auxin-dependent ubiquitination, followed by degradation, of proteins tagged with a specific auxin-inducible degron (AID) sequence [125]. This system was identified in plants and adapted to other species for the rapid auxin-induced degradation of the target protein [124,126,127]. In the work of McClure et al., a ubiquitously expressed Gal80 was fused with the AID sequence [124]. As a result, in the absence of auxin, Gal80 inhibits Gal4 activity and, consequently, the reporter gene’s expression is turned off. In the presence of the auxin phytohormone, which in low concentrations is non-lethal to both *Drosophila* larvae and adult flies, Gal80-AID is degraded and the Gal4 inhibition is released. This system was applied for the specific temporal and spatial control of the reporter gene’s expression and for the manipulation of *Drosophila* circadian locomotor rhythms [124].

#### 2.2.2. Chimeric Transcription Factors

To control the expression of endogenous genes, the fusion of different transcription factors with repression domains, resulting in their conversion to chimeric repressors, is often used. Here, we will review some examples of the application of chimeric repressor proteins in plants and animals.

#### Application of the Chimeric Repressors in Plants

In plants, for the silencing of endogenous gene expression, chimeric plant repressors are often used. For instance, the transcription factors Ethylene-insensitive3 (Ein3), Cup-shaped cotyledon1 (Cuc1), Production-of-anthocyanin-pigment1 (Pap1), and AtMyb23, which are involved in the responses to hormones, differentiation, the biosynthesis of metabolites, and development, were fused with the ERF-associated amphiphilic repression (EAR) motif [128]. Transgenic *Arabidopsis* plants expressing these chimeric Ein3, Cuc1, Pap1, and AtMyb23 repressors failed to respond to ethylene, had cup-shaped cotyledons, had a suppressed expression of genes related to phenylpropanoid biosynthesis, and had a reduced number of trichomes, respectively. Additionally, the chimeric AtMyb23 repressor was shown to regulate the target gene expression in epidermal cells, not only in leaves but also in the stems, roots, and seeds of *A. thaliana* plants [129].

The function of the MYB transcription factor AtMybL2 was studied via the fusion of the AtMybL2 protein with the SRDX repression domain [11]. As a result, it was demonstrated that AtMybL2 suppressed the expression of the *Dihydroflavonol 4-reductase* (*Dfr*) and *Transparent testa 8* (*Tt8*) genes and played a critical role in the regulation of anthocyanin biosynthesis. The fusion of the Gal4 DNA-binding domain with the AtMybL2 protein has shown that the AtMybL2 protein has a repression domain, TLLLFR, in its C-terminal region, which is different from the EAR repressor motif [11].

The tomato transcription factor Pti4, which belongs to the ethylene-responsive element-binding factors (ERFs), was fused with the AtHD2A protein to inhibit the expression of the GCC box-containing *GUS* reporter gene under transient expression assays in tobacco leaves [120].

A strategy for generating an effective chimeric repressor gene-silencing technology (CRES-T) in plants was described by Mitsuda et al. [130]. This tool, consisting of the fusion of a transcription factor to the plant-specific SRDX repression domain, can be used for the functional analysis of plant transcription factors. As a result, the transgenic plants with a chimeric repressor exhibit phenotypes similar to the loss-of-function alleles of the gene encoding the transcription factor. The CRES-T tool was successfully applied to *Arabidopsis* plants for the identification of transcription factors engaged in the salt, osmotic, and nitrogen deficiency stress response pathways [131,132]. Additionally, this tool was utilized for suppressing the B function to reduce floral organ identity in transgenic *Lilium* sp. plants [133].

#### Application of the Chimeric Repressors in Animals

To generate an estrogen-dependent repressor targeted to estrogen response elements-containing genes, a chimeric protein consisting of the estrogen receptor ERα and the KRAB repressor domain was constructed [134]. This chimeric repressor effectively inhibited the reporter *Luc* expression in ER-negative HepG2 human hepatoma cells in the presence of estrogen. The use of other promoters and cell lines has shown that the presence of estrogen response elements, rather than the capacity for estrogen induction, is responsible for the repression of the reporter gene by the ERα-KRAB repressor. Despite the fact that a single consensus estrogen response element was sufficient for repression, the repressor was unable to suppress transcription from the imperfect estrogen response element in the native pS2 promoter [134].

#### 2.2.3. Small Interfering Peptides (siPEPs)

One more approach used to regulate gene expression that uses transcription factors is the so-called small interfering peptides (siPEPs). Most transcription factors are known to act as homodimers or heterodimers and bind with different cofactors to form functional dimers [135,136]. Natural or synthetic small peptides can act as dominant negative regulators by forming heterodimers with transcription factors, which result in the disruption of endogenous transcription factor dimerisation and DNA binding (reviewed in [137]).

#### Application of siPEPs in Plants

siPEPs were suggested to be evolutionarily derived from functional transcription factors by the deletion of functional domains prior to the diversification of seed plants [138]. The potential siPEPs predicted in *Arabidopsis* were shown to have sequence similarities to members of major transcription factor families, such as basic helix-loop-helix (bHLH), homeobox, etc. [138]. Additionally, a group of genes encoding proteins with a small molecular size was found in *Arabidopsis* [139,140]. These small zipper (ZPR) proteins were shown to have a high sequence similarity to the class III homeodomain-leucine zipper transcription factors (HD-ZIP IIIs). However, they have only the ZIP motif, which mediates protein–protein interactions and lacks DNA-binding and activation domains. Since the HD-ZIP III transcription factors function as homodimers, these ZPRs were supposed to form non-functional heterodimers with the HD-ZIP III transcription factors. 

Other examples of siPEPs in plants include the regulation of the Auxin response factor (ARF) transcription factors by the Auxin/indole-3-acetic acid (Aux/IAA) proteins in auxin signalling [141,142,143,144], and the regulation of a group of zinc finger homeodomain (ZHD) transcription factors by Mini zinc finger 1 (MIF1) in growth hormone signalling [145,146].

Since transcription factors control the growth and development of plants, they seem to be promising targets for plant biotechnology, and siPEPs can be used for the repression of certain transcription factors to generate new features in crops.

#### Application of siPEPs in Animals

The ability of siPEPs to suppress the activity of transcription factors is actively used in the oncology field for the selective inhibition of major cancer oncoproteins (reviewed in [147]). Using siPEPs is a prospect for the development of novel cell-specific medicine tools, since siPEPs enable the blocking of selective protein–protein interactions that are difficult to target with conventional small-molecule chemicals.

### 2.3. Optogenetic Approaches

Optogenetics is an approach that combines genetic modifications and optical stimulation to regulate cellular processes [148]. The primary application of optogenetics is the use of light-sensitive ion channels to study neuronal activity [149]. However, the use of optogenetic approaches is not limited to studying neural networks. Optogenetic approaches utilising photoreceptors [150] allow for the control of the transcription of target genes using light [3,151,152,153,154]. The photoreceptors used in optogenetics can be divided into several groups: blue-light-utilising flavoproteins, cryptochromes, and xanthopsins; opsins utilising blue, green, and red light; and far-red- and near-infrared (NIR) light-sensing phytochromes [150]. The advantages of these optogenetic systems include focusing the light on a specific small area of the organism or cell; strict control of the intensity and duration of light exposure; the rapid initiation or termination of exposure by turning the light on or off, respectively; independence from the diffusion rate of a chemical agent; and no side effects that may arise from the use of chemical regulators (e.g., tetracycline) [155]. Optogenetic systems are used not only for the light-induced expression of genes of interest but also to suppress the expression of exogenous reporters and endogenous genes. Here, we will consider examples of the application of optogenetic systems to suppress gene expression in plants and animals.

#### 2.3.1. Application of Optogenetic Systems in Plants

The PULSE system facilitates the reversible control of gene expression in plants [156]. It is composed of a blue light repression module and a far-red light activation module. The repression induced by blue light is based on the photoreceptor EL222 from *Erythrobacter litoralis*, encompassing a light-oxygen voltage (LOV) and helix-turn-helix (HTH) domains. This protein forms dimers and binds to the target DNA sequence upon illumination with blue light [157]. In the PULSE system, EL222 was fused with the SRDX repression domain. When exposed to blue light, EL222-SRDX attaches to the promoter of the reporter gene, resulting in the repression of the target gene [156].

#### 2.3.2. Application of Optogenetic Systems in Animals

**Figure 5:**
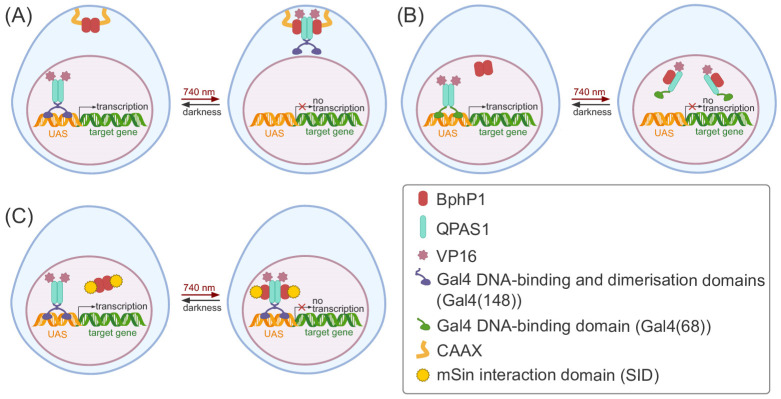
Near-infrared (NIR) light-induced repression of the reporter gene using a system based on the light-induced bacterial phytochrome photoreceptor 1 (BphP1) from *R. palustris* and its partner protein QPAS1. (**A**) Approach using the relocalisation of the activator to the plasma membrane. (**B**) Approach using the NIR light-induced “disruption” of the activator dimer. (**C**) NIR light-induced histone deacetylation of the reporter gene’s promoter region.

An optogenetic system based on the NIR light-induced heterodimerisation of the bacterial phytochrome BphP1 from *Rhodopseudomonas palustris* using its artificial partner protein QPAS1 was applied to inhibit the transcription of the reporter genes in HeLa cells, using several approaches [158]. The first approach was based on the NIR light-induced relocalisation of the chimeric transcription factor, consisting of the DNA-binding and dimerisation domains of Gal4 (Gal4(148)) fused with the QPAS1-VP16 construct, from the nucleus through the formation of heterodimers with BphP1 anchored to the plasma membrane (Figure 5A). The second approach was based on the NIR light-induced disruption of a chimeric homodimer consisting of the Gal4 DNA-binding domain (Gal4(68)) fused with the QPAS1-VP16 construct. In darkness, the protein Gal4(68)-QPAS-VP16 activated the expression of the reporter gene, forming functional dimers via the QPAS1 component. Under NIR light, QPAS1 interacted with BphP1, resulting in the disruption of the Gal4(68)-QPAS-VP16 homodimer and repression of the reporter gene (Figure 5B). In the last approach, BphP1 was fused with the mSin interaction domain (SID). The NIR light-triggered interaction of BphP1-SID with Gal4(148)-QPAS1-VP16 resulted in the recruiting of a mSin3-HDAC2 (histone deacetylase 2) complex to the reporter gene, followed by histone deacetylation and, consequently, the silencing of the gene of interest (Figure 5C) [158].

**Figure 6:**
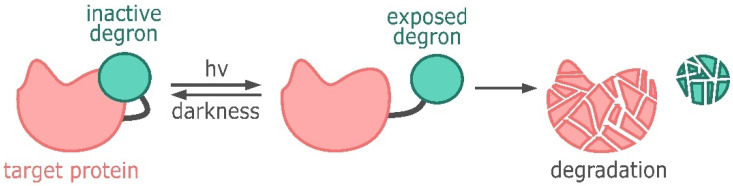
Regulation of the target protein level using light-induced degradation. In darkness the degron is not active, upon light induction the degron is exposed, leading to the degradation of the target protein.

There are also systems that control protein levels through light-induced degradation (Figure 6). The C-terminus of the photoresponsive Jα helix of the LOV2 domain of phototropin 1 from *Avena sativa* was fused with the RRRGN degron [159]. NIH3T3 cells expressing YFP fused to the LOV2 domain and the RRRGN degron displayed fluorescence when not illuminated. A significant decrease in fluorescence was observed in cells that were exposed to blue light (465 nm) for two hours. The system also demonstrated effective blue-light-induced degradation of the protein of interest in zebrafish embryos [159].

### 2.4. Zinc Finger Proteins (ZFPs)

C2H2-type ZFPs are DNA-binding proteins that interact with DNA in a modular and predictable manner. ZFPs constitute a diverse family of proteins, each containing one or more zinc finger domains, with zinc playing a pivotal role in domain stability. ZFPs are known to interact not only with DNA but also with RNA, lipids, and other proteins, facilitating their participation in various cellular processes such as chromatin remodelling, transcription regulation, signal transduction, etc. [160].

**Figure 7:**
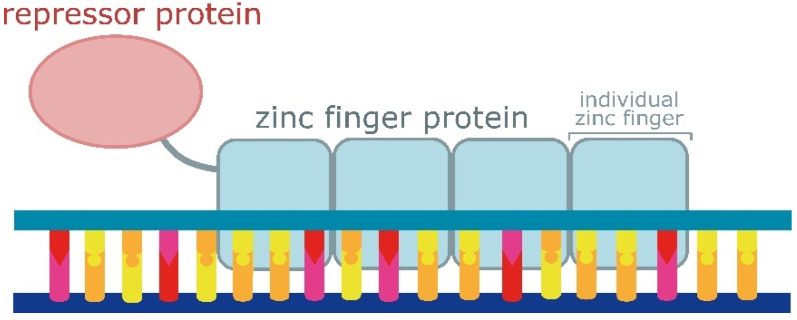
The structure of a zinc finger protein-based repressor protein. Zinc finger proteins are widely used for generating chimeric sequence-specific repressor proteins due to their modular structure. Each individual zinc finger touches 3 nucleotides.

The C2H2 zinc finger domain is one of the most common types of DNA-binding motifs. An individual zinc finger comprises approximately 30 amino acids in a conserved ββα configuration, stabilised by hydrophobic interactions and the coordination of a zinc ion with its cysteine and histidine residues [161]. Each zinc finger directly interacts with three consecutive nucleotides and one nucleotide of the reverse-complement strand within the adjacent trinucleotide [162]. On average, mouse and human ZFPs have about eight individual zinc fingers; hence, the average ZFP target motif is expected to comprise about 24 nucleotides [163]. The modular structure of ZFPs has rendered them attractive for designing sequence-specific DNA-binding proteins to activate or repress reporter gene expression (Figure 7). The key to utilising ZFPs for specific DNA recognitions has been the employment of more than three zinc finger domains. This progress was facilitated by the discovery of a highly conserved linker sequence, enabling the creation of chimeric variants containing polydactyl zinc finger proteins [164]. Thus, scientists have been able to identify DNA regions long enough to be unambiguously identified in the nucleotide sequence of entire genomes [165,166].

The main disadvantage of ZFPs is the difficulty of reprogramming them to target another sequence. Individual zinc fingers are very sensitive to their context; i.e., an individual zinc finger will provide affinity and specificity to a given triplet in one sequence but not in others, and an assembly of several zinc fingers does not always show the desired results [167,168]. Therefore, the selection of new zinc finger combinations is usually labour-intensive and does not guarantee success [169,170].

#### 2.4.1. Application of ZFPs in Plants

An artificial polydactyl ZFP-based repressor was designed to control the expression levels of transgenic and endogenous genes in *Arabidopsis* [171]. To achieve this, a ZFP specific to the *apetala3* (*ap3*) gene, responsible for the determination of floral organ identity, was fused with the SID repression domain. Most transgenic plants with ZFP-SID exhibited repressed expression levels of the *ap3* gene, with floral phenotypes similar to those of *ap3* and *sap* mutants. Additionally, the repression of the *GUS* transgene in the flowers of transgenic *Arabidopsis* plants with ZFP-SID expression was observed [171].

Transgenic *A. thaliana* lines expressing chimeric ZFPs fused with the KOX repressor demonstrated a repression of the *4-coumarate: coenzyme A ligase-1* (*At4cl1*) gene’s expression. At4CL1 is a key enzyme in lignin biosynthesis, and the downregulation of *At4cl1* may result in decreased lignin content, which is significant for the paper industry [172].

Another exciting application of ZFPs for gene regulation is the modulation of gene expression via targeted DNA methylation [173]. The recruitment of RNA polymerase V (Pol V) to an unmethylated epiallele *fwa-4* was achieved using a ZFP fused with a histone methyltransferase Su(var)3-9 homologue, the SUVH2 protein. As a result, DNA methylation extending approximately 150 bps in either direction from the ZFP-binding sites was observed, with the subsequent *fwa-4* gene silencing leading to a shortening of flowering time compared to the parental *Arabidopsis* line. 

#### 2.4.2. Application of Zinc Finger Proteins in Animals

Using a chimeric ZFP linked with the catalytic histone methyltransferase (HMT) domains of the SUV39H1 and G9A HMTs, the H3 lysine 9 methylation and consequent suppression of the endogenous *vascular endothelial growth factor A* (*VEGF-A*) gene’s expression were observed in HEK293 cells [174]. An increased transcriptional repression of the *VEGF-A* gene was observed when a chimeric ZFP fused with the v-ErbA repression domain, resulting in histone deacetylation, was recruited to the *VEGF-A* promoter in addition to ZFP-HMT. The methylation of 12 CpG sites in the *VEGF-A* promoter was also shown in human SKOV3 ovarian cancer cells using an artificial ZFP fused with the DNA methyltransferase Dnmt3a-3L [175].

Using DNA methyltransferase 3a (DNMT3a) fused with an engineered ZFP, it was possible to cause a stable and heritable downregulation of the tumour suppressor *Maspin* and the oncogene *SOX2* in breast cancer cells via the DNA methylation of the promoter regions of these genes [176]. Additionally, ZFPs were fused to the C-terminal catalytic domains of DNMT3a and DNMT3b to achieve the efficient methylation of the reporter promoter regions and consequent gene silencing in three different *Luc* reporter systems, including the *TK* (*Human herpesvirus 1 thymidylate kinase*) promoter, with an added binding sequence for Gal4 (UAS); the human *c-Ha-ras* gene promoter, with an added UAS; and the immediate early *IE175k* promoter of the Herpes Simplex Virus type 1 (HSV-1). It was shown that methylation-mediated gene silencing was effective in repressing infection with a wild-type HSV-1 [177].

The suppression of gene expression using ZFPs has also been carried out in a whole-organism model. Artificial ZFPs designed to bind long CAG repeats and fused with the KRAB domain were successfully used to repress the mutant *huntingtin* (*htt*) gene in STHdh cells, a mesothelial cell line from a heterozygous patient with Huntington’s disease (HD), and in the brain of R6/2 HD-model mice [178,179].

### 2.5. Transcription Activator-like Effectors (TALEs)

**Figure 8:**
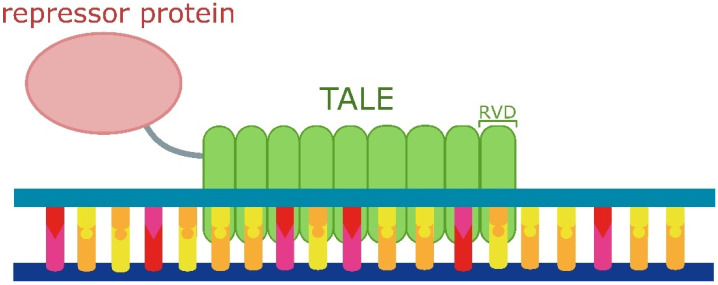
Schematic illustration of a transcription activator-like effector (TALE)-based repression system. One TALE repeat recognises one base pair by its repeat-variable di-residue (RVD).

Proteins similar to transcription activator-like effectors (TALEs) act as effectors that are secreted by *Xanthomonas* bacteria to aid in plant infection. They bind to specific DNA sequences and stimulate plant gene expression, promoting infection. TALEs consist of domains that determine DNA-binding specificity. One TALE repeat recognises one base pair by its repeat-variable di-residues (RVDs) with well-documented specificity [180], which is an advantage compared to zinc finger proteins [181]. Thanks to their modular structure and versatility TALEs have multiple applications in synthetic biology (Figure 8).

#### 2.5.1. Application of TALEs in Plants

A chimeric repressor consisting of the dHax3 [182] transcription activator-like effector (TALE) fused to the SRDX repression domain, targeting the *RD29A* gene promoter, was synthesised for the efficient repression of the *Luc* transgene and endogenous *RD29A* gene in stable transgenic lines of *Arabidopsis* plants [183]. Additionally, the *Arabidopsis* genome was scanned for potential dHax3 binding sequences. As a result, 70 possible targets of dHax3-SRDX in the genome of *A. thaliana* were identified, and several genes were under-expressed by at least 2-fold in all dHax3-SRDX transgenic plants [183].

#### 2.5.2. Application of TALEs in Animals

TALE repressors are designed to function in many eukaryotic systems. Most examples of repression are based on the fusion of TALEs to the KRAB domain [184]. TALEs recognising different target elements were fused with the KRAB domain and suppressed the expression of the fluorescent proteins AmCyan, mKate-PEST, and CFP in HEK293 cells [185,186]. It was also shown that the TALE-KRAB chimeric protein suppressed the expression of the exogenous reporter genes *GFP* and *Luc* in HEK293 and COS-7 cell lines, and effectively suppressed endogenous *TCF3* expression in a human B lymphoma cell line [187]. Moreover, combining TALE repressors with shRNAs targeting the same transcripts was shown to exhibit an almost complete repression of the *CFP* reporter gene in HEK293 cells [186].

There are also variants of TALE repressors utilising other repressive domains. The PIE-1 repression domain (PIE-1) from *C. elegans*, the QA domain within the *Ubx* gene (Ubx-QA) from *D. melanogaster*, the IAA28 repression domain (IAA28-RD) from *A*. *thaliana*, the SID, the Tbx3 repression domain (Tbx3-RD) and different truncations of the KRAB repression domain were used for generating TALE repressors targeting the promoter of the human *SOX2* gene [98]. The expression of these chimeric repressors in HEK293FT cells has shown that TALE-SID and TALE-KRAB enabled *SOX2* repression, while other repressors had little effect on *SOX2* expression [98]. Additionally, it was shown that, in mammalian (HEK293) cells, TALEs can exhibit repressor activity when binding to the sense strand in the transcribed region of the reporter gene, and this inhibitory effect is independent of the exact location and effector domain of the chimeric TALE protein [188].

The epiTALE system is a symbiosis of optogenetics and TALE [189]. The system consists of two parts: a TALE fused to the blue-light-sensitive cryptochrome 2 (Cry2) protein and different effector domains (SID, HDAC, HMTs, and acetyltransferase (HAT) inhibitors, as well as the HDAC and HMT recruiting proteins) fused to the Cry2 interacting partner CIB1 from *A. thaliana*. In the dark, TALE-Cry2 is located on DNA in the promoter region of the target gene, due to the specially selected TALE, and different effector domains fused with CIB1, in turn, float freely in the cell. Blue light induces the dimerisation of Cry2 and CIB1, thereby recruiting the effector domain to the target gene promoter. Using this system, 23 out of 24 epiTALEs successfully repressed the transcription of the *Grm2* gene in primary neurons and decreased *Neurog2* expression was observed in 20 of the 32 histone effector domains in Neuro 2a cells [189].

**Figure 9:**
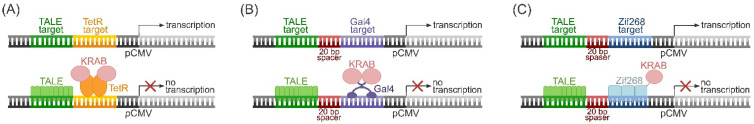
The effect of a transcription activator-like effector (TALE) binding on the inhibiting effect of chimeric repressor proteins. TALE binding leads to an augmentation of the inhibiting effect of tetracycline repressor (TetR) (**A**), galactose-responsive transcription factor Gal4 (**B**), and zinc finger protein (ZFP) Zif268 (**C**) transcription factors fused with the KRAB repressor domain.

Recently, it was shown that TALE binding leads to an augmentation of the inhibiting effect of other transcription factors, i.e., TetR, Gal4, and the ZFP Zif268 fused with the KRAB repression domain [190] (Figure 9). This effect was maintained regardless of the homodimeric or monomeric target transcription factor and seems to be due to TALE improving the binding characteristics at the transcription factor target site.

### 2.6. CRISPR/Cas-Based Transcription Regulation Systems

CRISPR (clustered regularly interspaced short palindromic repeats) is a family of DNA sequences found in the genomes of prokaryotic organisms such as bacteria and archaea [191]. These sequences play a key role in the antiviral (i.e., anti-phage) defence system of prokaryotes and provide a form of acquired immunity [191,192]. CRISPR is found in approximately 50% of sequenced bacterial genomes and almost 90% of sequenced archaeal genomes [193]. Cas (or “CRISPR-associated protein”) is an enzyme that uses CRISPR sequences as a guide to recognise and cleave specific DNA strands complementary to the CRISPR sequence. Cas enzymes, together with CRISPR sequences, form the basis of a technology known as CRISPR/Cas.

**Figure 10:**
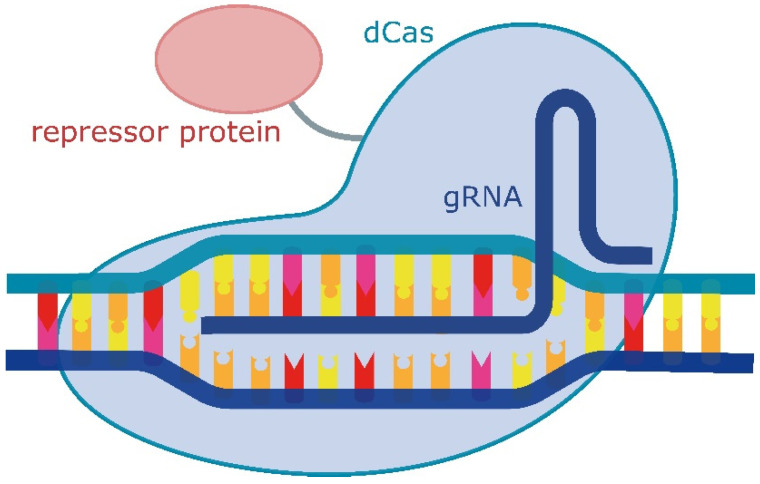
Schematic illustration of a CRISPR/Cas-based repression system.

The most widely used CRISPR-associated protein at the moment is the Cas9 protein from the bacterium *Streptococcus pyogenes* [194]. In recent years, CRISPR/Cas9 technology has evolved into a simple, effective, and precise method for modifying DNA [195,196]. This two-component system consists of the Cas9 nuclease, which, in complex with guide RNA (gRNA), forms double-strand breaks in DNA. Each gRNA molecule consists of three segments: a 20-nt long target-specific complementary region, a 42-nt Cas9-binding hairpin (Cas9 handle), and a 40-nt transcription terminator derived from *S. pyogenes* [197]. The specificity of the cleavage site is determined by a 20-nt sequence at the 5′ end of the gRNA and a 3-nt protospacer adjacent motif (PAM) sequence [198]. If the nuclease activity of the Cas9 protein is removed, it still remains capable of directed binding to DNA. This capability has made it possible to use the CRISPR/Cas9 system not only for genome editing but also as a platform for regulating gene expression. The Cas9 protein, which lacks endonuclease activity, i.e., catalytically dead Cas9 (dCas9), is used as a targeted DNA-binding complex to regulate the expression of genes of interest [197,199,200,201,202]. The fusion of dCas9 with their corresponding effector domains results in the robust activation (CRISPR activator, CRISPRa) or repression (CRISPR interference, CRISPRi) of transcription in prokaryotes and eukaryotes [197,203]. By targeting the gene at the promoter or coding sequence, the complex, with a repressor domain, interferes with the binding of RNA polymerase (Figure 10). Without the binding of RNA polymerase and transcription factors, the target gene’s expression is blocked [204].

The efficiency of the repression achieved by CRISPRi is influenced by many factors, the contributions of which are not yet well understood. For instance, information concerning the best location for a targeting site varies. For *Magnaporthe oryzae*, the most efficient targeting site position is 100–200 bp upstream of the transcription start site [203]. Similar results were observed using CRISPRi in *Saccharomyces cerevisiae* [205]. The optimal distance between the targeting site and transcription start site might be affected by the repressor domain of the CRISPRi system and its chromatin status [203].

**Figure 11:**
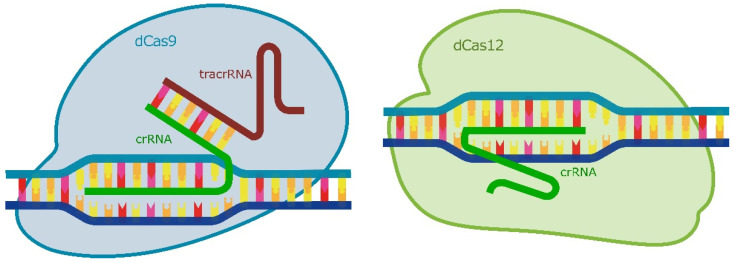
Comparison of the CRISPR/Cas9 and CRISPR/Cas12 systems. Short CRISPR-guide RNAs (crRNAs) are required for both Cas9 and Cas12 to function. However, Cas9 also requires the presence of trans-activating crRNA (tracrRNA).

A valuable advantage of CRISPRi is its capability for multiplexed gene suppression. For the multiplex regulation of gene transcription, the use of the dCas12 protein appears to be more convenient compared to the dCas9 protein. The type V endonuclease Cas12a (formerly Cpf1) is widely used for genome editing and generating diagnostic tools [206,207,208,209,210]. For a number of reasons, the Cas12 protein is better suited for transcription regulation than Cas9 (Figure 11). One of the distinguishing features of the Cas12 protein is its RNase activity, which it can use to cleave a long array of CRISPR RNAs (crRNAs) to generate individual crRNAs [210]. This enzyme also prefers T-rich PAMs (5′-TTTN-3′ or 5′-TTTV-3′; where V stands for A, C, or in some cases G), which enables it to be more readily recruited to AT-rich promoter sequences. This characteristic enables the simultaneous regulation of multiple genes, as well as the regulation of a single gene by recruiting Cas12 to different regulatory regions of the gene of interest. In addition to targeting different genes, its ability to use multiple gRNAs at once can be utilised to target different regions of the same gene to enhance transcriptional repression.

#### 2.6.1. Application of CRISPRi in Plants

The CRISPR/Cas system is widely used in plants not only for generating precise genomic mutations, but also for manipulating gene expression. In maize protoplasts, a combination of the chimeric protein dCas9-SRDX with gRNAs targeting different regions of the *ChlH* and *phytoene desaturase1* (*PDS1*) gene promoters showed a strong reduction in these genes’ expression. Additionally, a combination of two gRNAs showed a more effective reduction of the endogenous *PDS1* gene and the *Luc* reporter gene under the control of the *PDS1* promoter [211].

The dCas9 protein alone, and the chimeric dCas9-SRDX repressor in combination with three gRNAs designed to target and bind to the promoter region and the first exon of the *PDS* gene, were agroinfiltrated into leaves of *N. benthamiana*. As a result, it was shown that the strongest repression of the *PDS* gene was observed when the dCas9 and dCas9-SRDX proteins were guided by all three gRNAs simultaneously [212]. The use of dCas9-SRDX in *Arabidopsis* for repressing the *AtCSTF64* gene using multiple gRNAs is described in detail in the protocol of Lowder et al. [213].

In *Arabidopsis*, the activity of the dCas12 proteins from *Acidaminococcus* sp. BV3L6 (dAsCas12) and *Lachnospiraceae* bacterium ND2006 (dLbCas12), fused to three copies of the SRDX repressor domain, were compared [214]. As a result, the expression of the *miR159b* gene was less than 10% of the wild type in randomly chosen *Arabidopsis* transgenic lines with the dAsCas12-SRDX repressor. Similar repression activity, albeit with more variation, was observed in *Arabidopsis* transgenic lines with dLbCas12–SRDX.

Until recently, the SRDX repressor domain was the one most frequently used in plants. However, three more effective repressor domains, DLN144, DLS, and MIX, have been identified. The effective repression activity of these domains fused with dCas9 was shown in transiently transformed *N. benthamiana* leaves and in stable transgenic wheat and tobacco plants [215]. Two bacteriophage proteins, AcrIIA4 and AcrVA1, are known to inhibit CRISPR/Cas activity [216]. In *N. benthamiana*, these proteins blocked the CRISPR/Cas-based transcriptional activation of reporter genes in a highly efficient, dose-dependent manner [217].

#### 2.6.2. Application of CRISPRi in Animals

Using CRISPRi, it is possible to knock down both reporter and endogenous genes. For the effective silencing of transcription, a human codon-optimised dCas9 from *S. pyogenes* was fused with different repression domains including the KRAB domain, the cromo shadow (CS) domain of HP1α, and the WRPW domain of Hes1 [200]. The GFP+ HEK293 cells were transfected with these chimeric repressor proteins and a GFP-targeting gRNA. As a result, the dCas9-KRAB protein was the most effective, and cells expressing this protein showed a highly specific 5-fold and 15-fold decrease in GFP expression during transient and stable transfection, respectively. In HeLa cells stably expressing dCas9-KRAB, an efficient silencing (60–80% repression) of the transferrin receptor (*CD71*) and C-X-C chemokine receptor type 4 (*CXCR4*) endogenous genes was observed [200].

Also, the minimal CRISPRi system based on the dCas9 protein was successfully applied for the repression of the transcription of the long non-coding RNAs *roX1* and *roX2* [218]. This work showed that gRNAs targeting their non-template and template strands exhibited similar efficiencies of 95% and 85%, respectively. Furthermore, the results suggest that the use of multiple gRNAs at transcription start sites may enhance the efficiency of transcriptional silencing. The results were obtained on a model of the whole organism, *D*. *melanogaster*.

## 3. Concluding Remarks

Controlling gene expression requires developing approaches that allow for the precise recognition of specific DNA sequences within the context of the genome. Over recent years, several methods for targeted gene silencing have been developed. RNAi, enabling temporal gene silencing by knocking down target mRNA transcripts, is widely employed in both animals and plants. This rapid and straightforward method is useful for studying essential gene functions. However, one of the principal disadvantages of RNAi is the presence of off-target effects (Table 1), which are critical in large-scale studies that involve silencing large numbers of genes in parallel [219]. Additionally, RNAi is not always highly efficient in knocking down target genes. Unlike RNAi, optogenetic approaches are much less frequently used for directed gene silencing. The use of optogenetic approaches is especially challenging in plants, since ambient light, necessary for the normal growth and development of plants, can cause the non-specific activation of optogenetic systems. Other approaches utilising fusions of DNA-binding proteins (ZFPs, TALEs, chimeric transcription factors, and CRISPRi) with repressive domains can affect nearby genes depending on the repressor domain used, and that sometimes leads to unexpected adverse effects (Table 1). Additionally, CRISPRi and TALE appear to be more efficient compared to synthetic ZFPs, and CRISPRi is easier to modify, as it does not require laborious cloning to obtain site-specific DNA-binding proteins, unlike ZFP- or TALE-based approaches. However, the application of CRISPRi in plants has some limitations, since recently it was shown that guide RNA caused Cas-independent gene silencing in three plant species, including tobacco *N*. *benthamiana*, tomato *Solanum lycopersicum*, and stable transgenic *A. thaliana* [220]. Of the CRISPRi processes, the use of the dCas12 protein is currently the most promising, as it can enable the targeting of several regions of the gene simultaneously, which can increase the efficiency of inhibiting the target gene’s expression. Additionally, multiplex gRNAs may be used for the simultaneous targeting of several genes, which may be useful in the study of signalling pathways, including the regulation of the production of desired metabolites [221,222]. 

## Figures and Tables

**Table 1:** Comparison of RNAi, Gal4/UAS, chimeric transcription factors, optogenetic systems, ZFPs, TALEs, and CRISPRi-based technologies.

Feature	RNAi	Gal4/UAS	Chimeric TFs	Optogenetic Systems	ZFPs	TALEs	CRISPRi
Off-target effects’ probability	high	low	low	low	medium	low	low
Off-target space	transcriptome	nearby genes	nearby genes	nearby genes	genome, nearby genes	nearby genes	genome, nearby genes
Ease of experiment design	easy	easy	moderate	moderate	difficult	difficult	moderate
Repression efficiency	often low in animals	high	high	high	high	high	high
Ability to repress multiple genes	moderate	low	low	low	low	low	high
Application in plants	possible	possible	possible	has limitations due to nonspecific activation under ambient light	possible	possible	has limitations due to gRNA-mediated Cas-independent gene silencing
Application in animals	has a risk of immune response	possible	uncontrollable activity of chimeric TFs may cause adverse effects	has limitations in whole organisms due to low penetration depth of short-wavelength light	possible	possible	possible

RNAi—RNA interference, TFs—transcription factors, ZFPs—zinc finger proteins, TALEs—transcription activator-like effectors, CRISPRi—CRISPR interference.

## Data Availability

Not applicable.
